# Regular Eating

**Published:** 1873-10

**Authors:** 


### REGULAR EATING.

HALF of all ordinary diseases would be banished from civilized life, and dyspepsia become almost unknown, if everybody would eat but thrice a day at regular times, and not an atom between meals, the intervals being not less than five hours, that being the time required to digest a full meal and pass it out of the stomach.

If a person eats between meals the process of digestion of the food already in the stomach is arrested, until the last which has been eaten is brought into the condition of the former meal, just as if water is boiling and ice is put in, the whole ceases to boil until the ice has been melted and brought to the boiling point, and then the whole boils together.

But it is a law of nature that all food begins to decay, to rot, after exposure to heat and moisture for a certain time. If a meal is eaten, and in two hours another, the whole remains undigested for seven hours, before which time the rottening process commences, and the man has his stomach full of carrion—the very idea of which is horribly disgusting; but that such is the case the unendurable odor of the belchings demonstrate.

As, then, all the food in the stomach is in a rotting condition, in a state of fermentive decay, it becomes unfit for the purposes of nutrition and for making good pure blood.

Small wonder is it that dyspeptics have such a variety of symptoms, and aches, and complaints in every part of the system, for there is not one drop of pure blood in the whole body; hence the nerves, which feed on this impure and imperfect blood, are not properly nourished, and, as a consequence, become diseased. They "complain;" they are hungry—and like a hungry man are peevish, fretful, restless. We call it nervousness, and no one ever knew a dyspeptic who was not restless, fretful, fidgety, and essentially disagreeable, fitful and uncertain.

The stomach is made up of a number of muscles, all of which are brought into requisition in the process of digestion. But no muscle can work always. The busy heart is in a state of perfect repose for one third of its time. The eye can work twice in a second, but this could not be continued five minutes. The hands and feet must have rest, and so with the muscles of the stomach; they only can rest when there is no work for them to do—no food in the stomach to digest. Even at five hours' interval, and eating thrice a day, they are kept constantly at work from breakfast until the last meal is disposed of, usually ten o'clock at night. But multitudes eat heartily within an hour of bed time; thus, while the other portions of the body are at rest, the stomach is kept laboring until almost daylight, and made to begin again at breakfast time. No wonder is it that the stomach is worn out—has lost its power of action. Many girls become dyspeptic before they are out of their teens, in consequence of being about the house and nibbling at everything they lay their eyes on that is good to eat.

